# Identification of metastasis-associated protein 1 (MTA1) as a new molecular marker for canine urothelial carcinoma

**DOI:** 10.3389/fvets.2025.1527167

**Published:** 2025-04-25

**Authors:** Gisella Campanelli, Noah Waxner, Nema Parkhomovsky, Chun Kuen Mak, Ji-Hang Yin, Susanne Je-Han Lin, Raphael Vanderstichel, Ching Yang, Anait S. Levenson

**Affiliations:** ^1^Department of Veterinary Biomedical Sciences, Lewyt College of Veterinary Medicine, Long Island University, Brookville, NY, United States; ^2^College of Sciences, Long Island University, Brookville, NY, United States; ^3^Department of Veterinary Clinical Sciences, Lewyt College of Veterinary Medicine, Long Island University, Brookville, NY, United States; ^4^Department of Pathobiology, College of Veterinary Medicine, Auburn University, Auburn, AL, United States; ^5^Department of Veterinary Pathology, College of Veterinary Medicine, Iowa State University, Ames, IA, United States

**Keywords:** canine urothelial carcinoma, canine cell lines, immunohistochemistry, MTA1, COX2, E-cadherin

## Abstract

**Background:**

Although metastasis-associated protein 1 (MTA1) is known to play a role in cancer invasion and metastasis of various cancers, the clinical significance of its expression in canine urothelial carcinoma (UC) has not been explored. We sought to evaluate the expression of MTA1, cyclooxygenase 2 (COX2) and E-cadherin (E-cad) in association with clinicopathological parameters in clinical samples of canine UC.

**Methods:**

We retrospectively analyzed UC tissues from 28 canine patients using immunohistochemistry for Ki67, CD31, MTA1, COX2, and E-cad staining. Statistical significance for marker staining intensities was evaluated by ANOVA or Student’s *t*-test. The correlation between molecular markers in canine UC samples detected by IHC and clinicopathological features was calculated by the Wilcoxon (Mann–Whitney) and Kruskal-Wallis tests. Western blot analysis was performed for detection of EMT markers in canine cell lines.

**Results:**

We show that MTA1 and COX2 are overexpressed in canine UC samples compared to normal canine bladder samples, whereas E-cad levels are higher in normal bladder. The results demonstrated that MTA1 expression correlated with aggressive clinicopathological features such as high tumor-grade, muscular/vascular invasion, and metastasis. The expression of MTA1 differed in tumors depending on their localization, with the highest being in the urethra adjoining the prostate. Unexpectedly, higher E-cad levels were detected in metastatic tumor cells compared to primary tumor cells.

**Conclusion:**

These findings suggest that MTA1 may represent a key upstream effector tightly associated with COX2 and E-cad-mediated events in canine UC. Accordingly, MTA1 may be considered a feasible interceptive and therapeutic target for canine UC treatment.

## 1 Introduction

Canine urothelial carcinoma (UC), previously known as transitional cell carcinoma, is an aggressive malignancy, having an estimated occurrence of over 60,000 new cases in the United States each year (1). By the time of diagnosis, most canine UC is already characterized by high histologic grade and muscular/vascular infiltration, with distant metastases detectable in about 20% of cases (2). Currently, systemic drug therapy is the major treatment approach, which is often associated with high toxicity (3). To advance the development of more clinically valuable targeted therapies, various molecular markers implicated in canine UC including EGFR/ HER2, PI3K/AKT/mTOR, RTK/RAS, PD-1, and cyclooxygenase 2 (COX2) among others, have been recognized as potential targets (3–5). Moreover, some of these targeted therapies (i.e., COX2 inhibitors) were effective as single agents and demonstrated enhanced chemotherapy activity when used in combined settings (6–9). Despite this, a need remains for a better understanding of the molecular features of canine UC to identify new targets for efficacious novel therapies.

Metastasis-associated protein 1 (MTA1) is an epigenetic reader and transcriptional regulator. Overexpression of MTA1 is strongly associated with more aggressive tumor behavior, advanced stages, metastasis, and an overall poor prognosis in a range of different cancers (10–13). Metastasis-associated protein 1 plays an important role in human prostate cancer (10, 14, 15), but much less is known about its importance in canine and human bladder cancer. Only two studies focusing on MTA1 in human bladder cancer are present in the literature, one considering significantly high MTA1 expression in tumor tissues as a potential target (16) and the other demonstrating MTA1-mediated apoptosis and other antitumor effects in response to the natural compound *β*-elemene, found in *Curcuma Rhizoma* (17). Naturally occurring UC in dogs resembles human bladder cancer in many clinicopathologic characteristics. However, unlike in humans, canine UC usually presents more aggressively with high-grade muscle invasion encompassing more than 90% of cases (5), which can be associated with unidentified altered molecular pathways. To date, the expression of MTA1 has not been studied in veterinary medicine; thus, the current study aimed to investigate the MTA1 expression in canine UC and to determine a possible relationship between MTA1 expression and epithelial-to-mesenchymal transition (EMT)-related events associated with invasiveness and enhanced migratory capacity (18). The major molecular event of EMT is a “cadherin switch” in which a downregulation of a junctional protein E-cadherin (E-cad) results in loss of cell–cell adhesion (19), and a *de novo* expression of N-cadherin, which signifies malignant transformation and increased migratory capacity (20). In addition, a few regulatory proteins have been identified as EMT inducers, among them is COX2, high levels of which have been detected in greater than 80% of canine UC cases (21–23). Importantly, several studies have reported that selective COX2 inhibition leads to reduction of EMT in human bladder cancer (24–26). While MTA1 has been shown to play a role in regulating EMT (27–30) and negatively controlling E-cad expression in humans (31–36), further work is needed to elucidate the role of MTA1 and its link to EMT in canine invasive UC.

The goals of the current study were: first, to evaluate the expression of MTA1, COX2, and E-cad in clinical samples of canine UC and metastatic lesions, and to compare these to normal bladder tissues; second, to correlate the pattern of MTA1 expression with other markers and clinicopathological parameters; and third, to examine MTA1 expression and EMT-related markers in canine UC cell lines in order to establish a foundation for further functional and mechanistic studies.

## 2 Materials and methods

### 2.1 Samples and histology

For the current study, clinical samples were obtained from 23 cases of canine UC and 5 normal canine bladders as controls. Samples were received from the tissue archives of Long Island University, Auburn University, and Iowa State University. All tissue sections were confirmed to be UC by pathologists (JHY, SJL, and CY). General information and clinicopathological data of these cases is provided in Table 1. A total of 10 (43.5%%) out of 23 were male dogs (all of them neutered), while 13 (56.5%) were female dogs (spayed in 11 cases). The age of the dogs ranged from 9 to 17 years with an average of 13.4 years for males and 12.4 years for female dogs. Breeds were represented as follows: mixed breed dogs (*n* = 6, 26.1%), Terriers (*n* = 4, 3.13%), Labrador Retrievers (*n* = 2, 8.7%), Golden Retriever (*n* = 1,4.3%), Shetland Sheepdog (*n* = 1, 4.3%), Beagle (*n* = 1, 4.3%), Bichon (*n* = 1, 4.3%), Bichon Frise (*n* = 1, 4.3%), Lhasa Apso (*n* = 1, 4.3%), German Shorthair Pointer (*n* = 1, 4.3%), Tibetan Spaniel (*n* = 1, 4.3%), Pembroke Welsh Corgi (*n* = 1, 4.3%), Chihuahua (*n* = 1, 4.3%), Miniature Dachshund (*n* = 1, 4.3%). Eleven cases involved metastasis to different organs: lung (*n* = 7, 29.2%), liver (*n* = 4, 16.7%), lymph nodes (*n* = 3, 12.5%), brain (*n* = 1, 4.2%), dura mater (*n* = 1, 4.2%), adrenal gland (*n* = 2, 8.3%), kidney (*n* = 1, 4.2%), heart (*n* = 1, 4.2%), jejunum (*n* = 1, 4.2%), and subcutis (ventral abdomen) (*n* = 1, 4.2%). Normal cases consisted of male (*n* = 4, 80.0%) and female (*n* = 1, 20.0%) dogs; all normal cases were neutered; average age: 8.8 years; breeds: mixed (*n* = 2, 40.0%), Terrier (*n* = 1, 20.0%), Pomeranian (*n* = 1, 20.0%), and German Shepherd (*n* = 1, 20.0%).

**Table 1:** Clinicopathological characteristics of 23 cases of canine urothelial carcinoma.

Case No.	Breed	Sex^a^	Age (Year)	Distant metastasis	Mitotic count per 2.37 mm^2^	Lamina propria invasion	Muscular invasion	Vascular invasion	Meuten grade (Low/High)	Valli grade (1–3)
1	Mixed	MC	14	Yes	14	Yes	Yes	Yes	High	3
2	Mixed	MC	9	Yes	13	Yes	Yes	Yes	High	3
3	Mixed	FS	9	Yes	17	Yes	No	No	High	3
4	Collie	FS	11	Yes	6	Yes	Yes	No	High	3
5	Pembroke Welsh Corgi	FS	11	Yes	21	Yes	Yes	Yes	High	3
6	Beagle	FS	13	Yes	6	Yes	Yes	Yes	High	3
7	German Shorthair Pointer	F	15	Yes	10	Yes	Yes	Yes	High	3
8	Tibetan spaniel	MC	15	Yes	6	Yes	Yes	Yes	High	3
9	Golden Retriever	FS	11	Yes	23	Yes	Yes	Yes	High	3
10	Bichon	MC	16	Yes	16	Yes	No	No	High	3
11	Lhasa Apso	FS	14	Yes	19	Yes	Yes	Yes	High	3
12	Mixed	MC	12	No	24	Yes	Yes	Yes	High	3
13	Mixed	MC	14	No	41	Yes	Yes	No	High	3
14	Mixed	MC	15	No	4	Yes	N/A	N/A	High	3
15	Cairn Terrier	MC	12	No	14	Yes	Yes	No	High	2
16	Jack Russel Terrier	FS	13	No	24	Yes	Yes	No	High	3
17	Maltese	MC	14	No	27	Yes	No	No	High	3
18	Labrador Retriever	F	14	No	67	Yes	Yes	Yes	High	2
19	Labrador Retriever	FS	10	No	8	Yes	Yes	No	High	3
20	Shetland sheepdog	FS	9	No	21	Yes	No	No	High	3
21	Miniature Dachshund	FS	17	No	23	Yes	No	No	High	2
22	Chihuahua	FS	14	No	11	Yes	Yes	Yes	High	3
23	Bichon Frise	MC	13	No	1	Yes	No	Yes	High	2

a MC: castrated male; F: female; FS: spayed female.

N/A: not available due to the lack of muscularis externa and adventitia or serosa for evaluation in the section examined.

All full thickness biopsies had been fixed in 10% buffered formalin and embedded in paraffin. Slides were stained with hematoxylin–eosin (H&E) for histological evaluation and probed for molecular markers by IHC.

Tumors were graded according to the World Health Organization 2004 guidelines of domestic animal tumors and based on the grading systems for canine UC by Valli et al. (37) and Meuten (2). In addition, mitotic count and invasion of the lamina propria, muscle and vessels were recorded.

### 2.2 Immunohistochemistry

Slides were subjected to IHC analysis as described previously (38). Briefly, tissues were deparaffinated, hydrated, and treated to expose target proteins. Antigen retrieval for each target was undertaken using Antigen Unmasking Solution, citric acid based (Vector Labs, H-3300). Active sites were blocked with serum (normal goat or horse) and endogenous peroxidases were quenched with 3% hydrogen peroxide. Tissues were then incubated in primary antibody [Ki67, CD31, MTA1, COX2, and E-cad antibodies were used (Supplementary Table S1)] overnight at 4°C. Respective secondary antibody (for Rabbit VectaStain Kit, biotinylated goat anti-rabbit antibody; for Mouse VectaStain Kit, biotinylated horse anti-mouse antibody) and avidin-biotin complex were used as per manufacturer’s protocol (Vectastain Elite ABC-HRP Kit, Vector Laboratories, Newark, CA, United States). Stain was developed using ImmPACT DAB Substrate Kit (Vector Laboratories, Newark, CA, United States) and nuclei were counterstained using hematoxylin. Tissues were then dehydrated, cleared with xylene, and mounted with glass coverslips. Immunostaining images were taken using an EVOS XL Core microscope (Thermo Fisher Scientific, Somerset, NJ, United States). Five random fields per case were selected for quantitation. ImageJ software (NIH, Bethesda, MD, United States) was used for counting Ki67-, MTA1-, COX2-, and E-Cad -stained cells and for measuring CD31 endothelial-stained vessel area. MTA1-stained nuclei, E-cad-stained membranes, and COX2 cytoplasmic staining were scored 1–3, where 1 (+) was assigned to weak staining intensity, 2 (++) for moderate staining intensity, and 3 (+++) for strong staining intensity. Staining intensity was represented graphically against frequency. The proportion and intensity scores were then multiplied to obtain a total stain score.

### 2.3 Cell culture

Canine invasive transitional cell carcinoma (UC) cells lines K9TCC-PU-AxA (AxA), K9TCC-PU-Sh (Sh), K9TCC (Org), and K9TCC-PU- Nk (Nk) were generous gifts from Dr. Deborah Knapp, Purdue University, IN. Cells were cultured in DMEM/F12 media with 10% fetal bovine serum and maintained at 37°C with 5% CO_2_. Cells were regularly tested for mycoplasma using the Universal Mycoplasma Detection Kit (ATCC) and found to be mycoplasma-free.

### 2.4 Western blot analysis

Western blots were performed as described previously (38). Briefly, protein lysates were prepared from a confluent 100 mm cell culture plate using RIPA buffer (Thermo Fisher Scientific, Somerset, NJ, United States). Protein concentration was estimated, and 30 μg of protein was loaded and separated using 10–15% sodium dodecyl sulfate-polyacrylamide gel electrophoresis followed by transfer to a polyvinylidene difluoride membrane (Immun-blot, Bio-Rad, Hercules, CA, United States). Membranes were blocked with 5% milk/TBS/0.1% Tween 20 for 1 h. Subsequently, membranes were probed overnight with corresponding primary antibodies listed in Supplementary Table S1. After treatment with respective secondary antibody (goat anti-rabbit, HRP-linked 1 mg/mL; goat anti-mouse, HRP-linked 1 mg/mL (Sigma-Aldrich); 1:2,500), signals were developed using enhanced chemiluminescence (Thermo Fisher Scientific, Somerset, NJ, United States) and detected on ChemiDoc Imaging System (Bio- Rad, Hercules, CA, United State). *β*-actin was used as a loading control. Band intensity was measured using Image J (NIH, Bethesda, MD, USA).

### 2.5 Statistical analysis

Quantitative data are represented as the mean ± SEM. Statistical significance for marker staining intensities was evaluated by ANOVA or Student’s *t*-test using GraphPad Prism v9 software. Additionally, statistical analyses of the association between markers and clinicopathological parameters were conducted using the Wilcoxon (Mann–Whitney) test for non-parametric comparisons between two groups and the Kruskal-Wallis test for comparisons among three or more groups. For within-subject comparisons (e.g., primary versus metastatic tumors), the Wilcoxon matched-pairs signed-rank test was applied. Continuous predictors were evaluated through linear regressions, ensuring the assumptions of normality and homoscedasticity, with trends visually represented using locally-weighted scatterplot smoothing (bandwidth = 0.8). All analyses were performed using Stata v18 (StataCorp, College Station, TX, United States), with *p*-values less than 0.05 considered statistically significant.

## 3 Results

### 3.1 MTA1 expression in canine clinical UC samples and metastatic sites compared to normal control bladders

**Figure 1:**
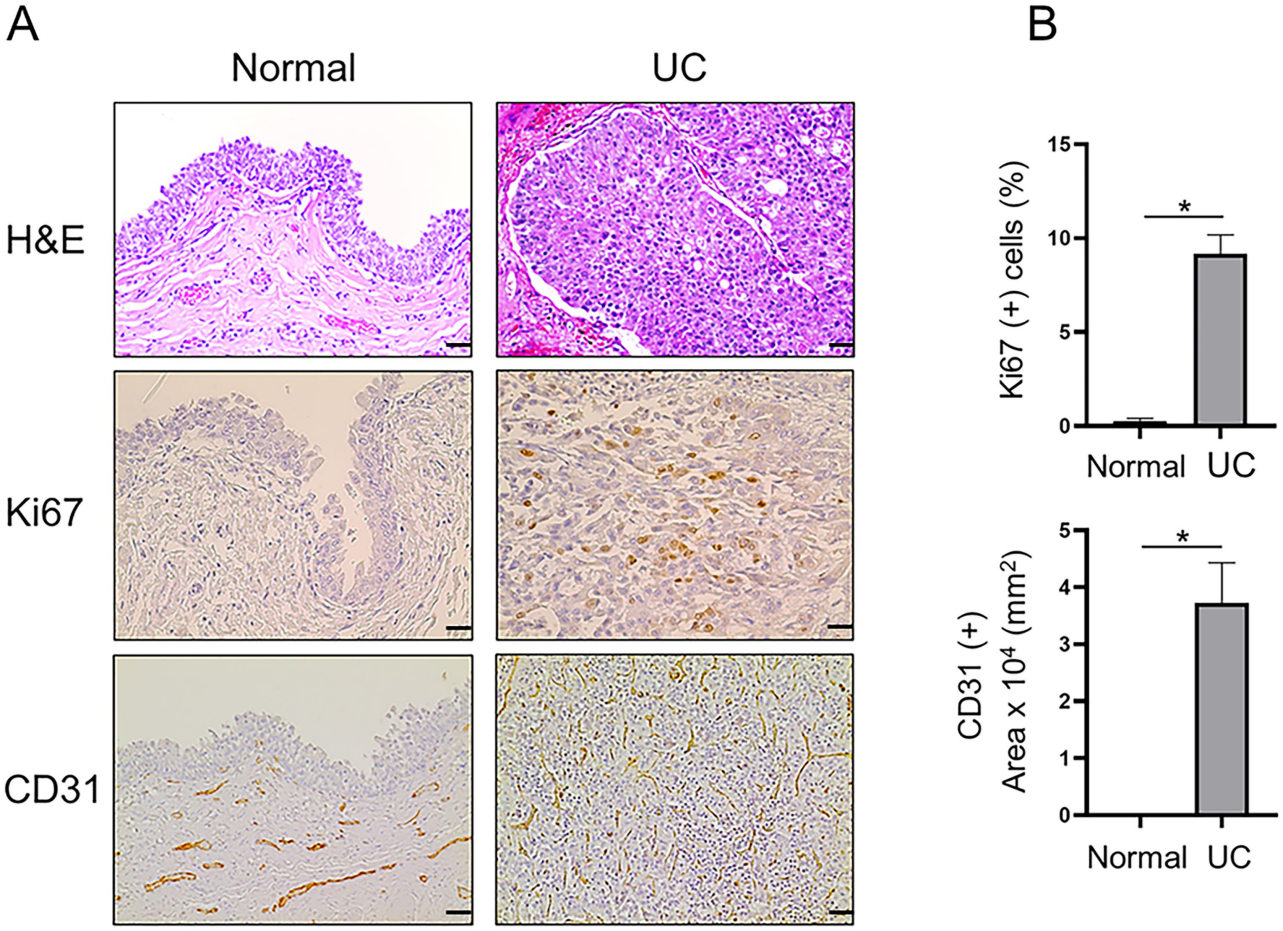
**(A)** Representative H&E and IHC staining demonstrating the histopathology and expression of Ki67 (scale bar: 20 μm) and CD31 (scale bar: 50 μm) in canine normal and UC tissue samples. **(B)** Quantitative analysis of IHC staining for each marker. Note re CD31 staining: For normal bladder, there are vessels in the lamina propria but not in the urothelial layer (3rd panel, *left*). For tumor, vessels grow in between the urothelial cells (3rd panel, *right*). Values are mean ± SEM of positive cells counted in five randomly selected fields per sample for *n* = 5 (normal) and *n* = 23 (UC). *p ≤ 0.05 (Student’s *t*-test).

**Figure 2:**
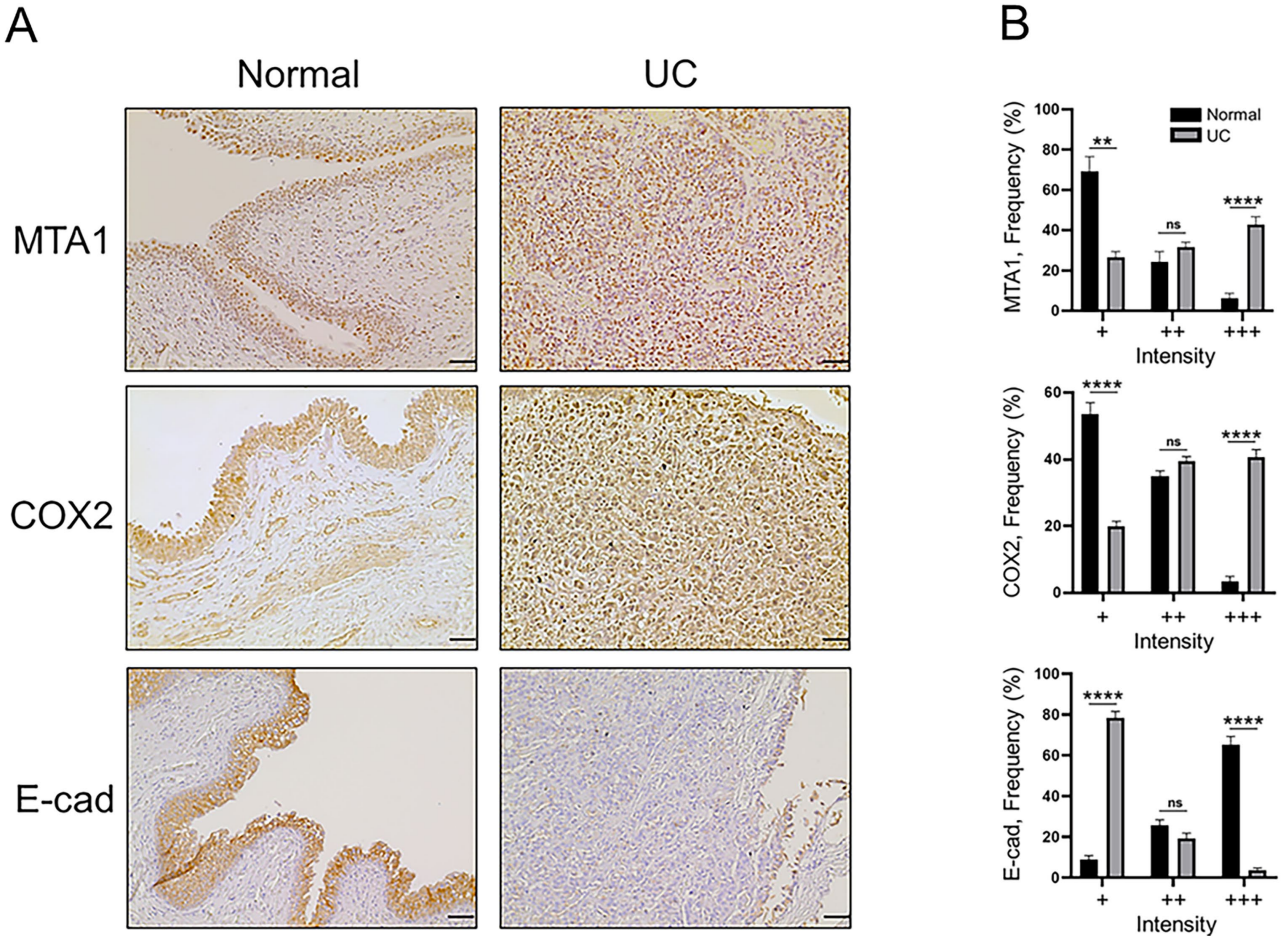
**(A)** Representative IHC staining of canine normal and UC tissue samples demonstrating the expression of MTA1, COX2, and E-cad (scale bar: 50 μm). **(B)** Quantitative analysis of IHC staining for each marker. Values are mean ± SEM of positively stained cells counted in five randomly selected fields per sample for *n* = 5 (normal) and *n* = 23 (UC). **p < 0.01; ****p < 0.0001; ns, non-significant (Multiple Student’s *t*-test).

Immunohistochemical staining was performed on 23 UC and 5 normal bladder tissue samples to analyze the differences in Ki67 (proliferation marker), CD31 (angiogenesis) and the molecular markers MTA1, COX2, and E-cad. The urothelium of control bladders was negative for Ki67, while neoplastic urothelial cells exhibited high levels of Ki67 nuclear staining, indicating a high proliferative status (Figure 1). CD31 was only observed in the lamina propria, submucosa, and adventitia or serosa in the control bladder tissues but not in the urothelium. In contrast, extensive neovascularization (angiogenesis) was detected in the neoplastic urothelium (Figure 1). Furthermore, MTA1, COX2, and E-cad expressions were detected in both normal bladder and UC tissues, however with different patterns of frequency and intensity (Figure 2). The majority of normal urothelium in control bladder tissues demonstrated weak (+) MTA1 nuclear (69.4%). Interestingly, we detected mild COX2 cytoplasmic (53.6%) staining in normal urothelium in contrast to that found by Khan et al. (39), yet in line with data showing two different patterns of COX2 expression in hyperplastic urothelium (4). Conversely, tissues from UC samples demonstrated significantly greater frequency of cells with strong staining intensity (+++) for MTA1 (42.9% vs. 6.3%, *p* < 0.0001) and COX2 (40.7% vs. 3.6%, *p* < 0.0001) compared to normal urothelium. The results indicated a high expression of both markers throughout the neoplastic cell population. On the other hand, normal urothelial cells showed significantly higher strong staining intensity (+++) for E-cad compared to UC samples (65.3% vs. 3.7%, *p* < 0.0001) (Figure 2).

**Figure 3:**
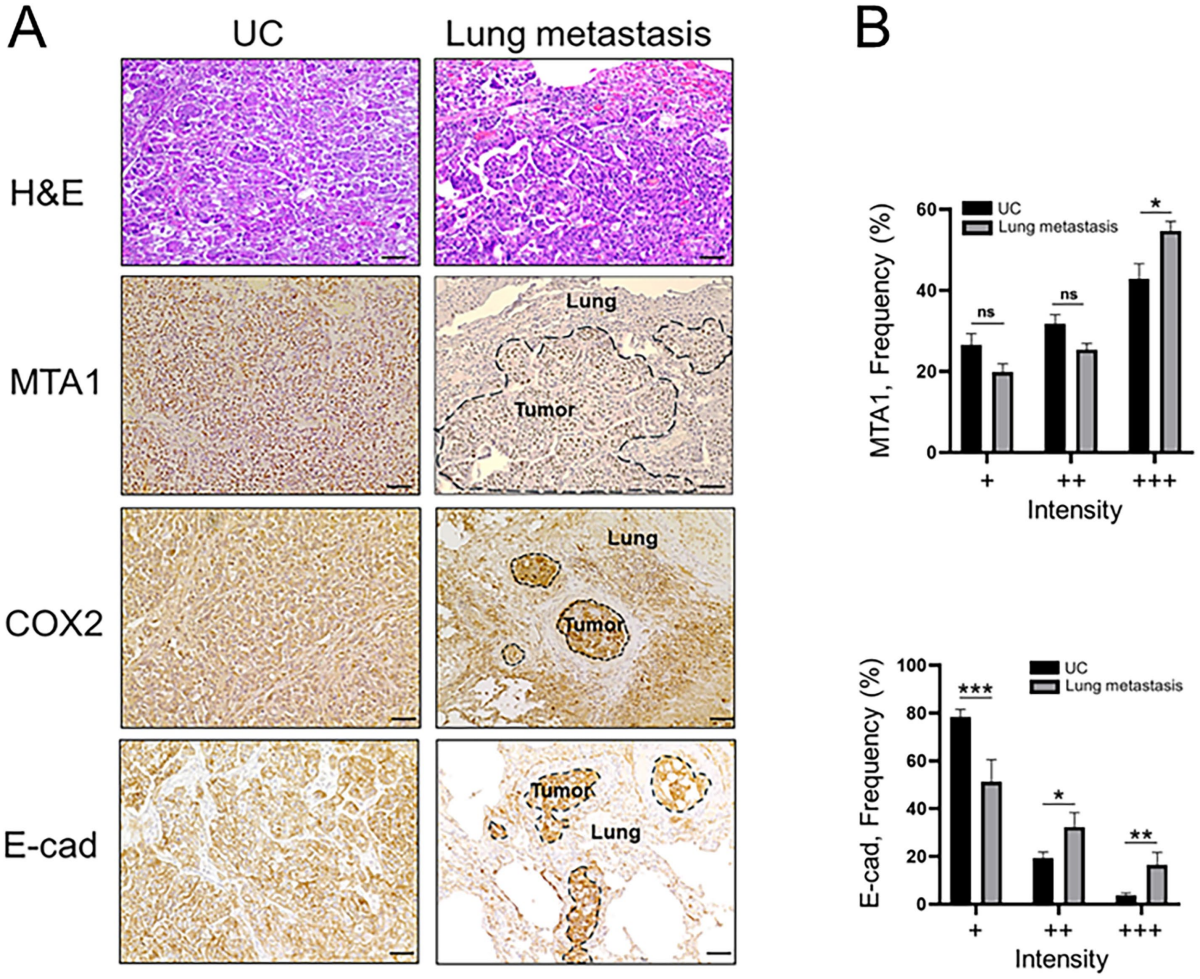
**(A)** Representative H&E and IHC staining demonstrating the histopathology and expression of MTA1, COX2, and E-cad in canine primary tumors (UC) and lung metastases (scale bar: 50 μm). **(B)** Quantitative analysis of IHC staining for MTA1 and E-cad expression. Values are mean ± SEM of positively stained cells counted in five randomly selected fields per sample for *n* = 23 (UC) and *n* = 11 (metastasis). *p < 0.05; **p < 0.01; ***p < 0.001; ns, non-significant (Multiple Student’s *t*-test).

Immunohistochemical analyses of canine lung metastatic tissues revealed a significantly greater staining intensity (+++) for MTA1 compared to primary tumor tissues (53.2% vs. 42.9%, *p* < 0.05) (Figure 3). COX2 was also found to have greater staining intensity in lung metastases compared to primary tumor. Curiously, metastatic tissues showed a significantly greater strong staining intensity (+++) for E-cad compared to primary tumor tissue (16.5% vs. 3.7% *p* < 0.01) but less than normal tissues (Supplementary Figure S1).

### 3.2 Relationship between MTA1 expression and clinicopathological characteristics in canine UC

**Figure 4:**
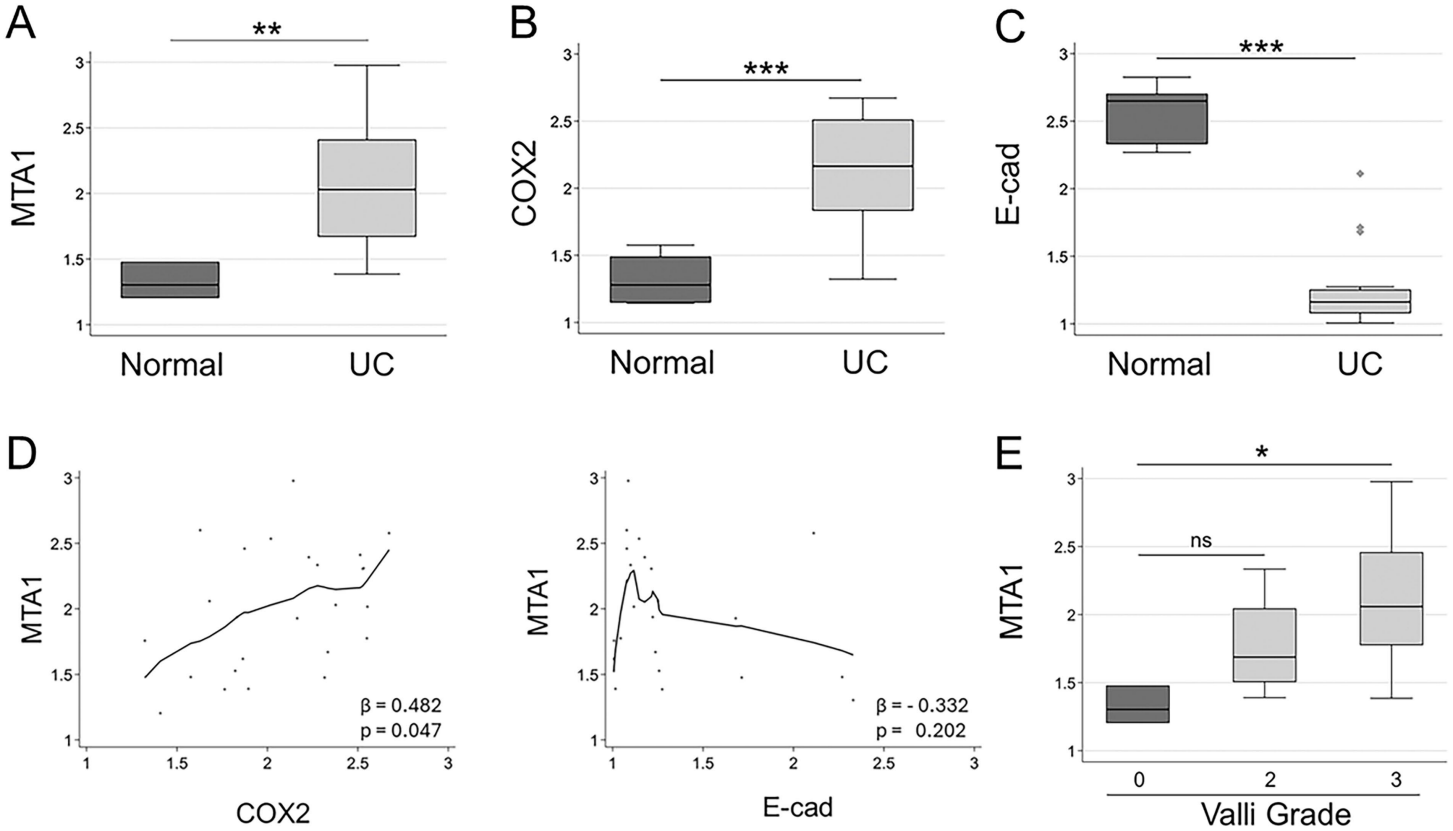
**(A)** MTA1 **(B)** COX2, and **(C)** E-cad expression in clinical samples of canine UC (*n* = 23) compared to normal bladder tissues (*n* = 5). **p < 0.01; ***p < 0.001, Wilcoxon (Mann–Whitney) test. **(D)** *Left*, Locally-weighted scatterplot showing a positive linear relationship between MTA1 and COX2 (*β* = 0.482; *p* ≤ 0.05), *Right,* Locally-weighted scatterplot showing the inverse relationship between MTA1 and E-cad (*β* = −0.332; ns). **(E)** MTA1 expression in normal tissues and tumors with different Valli grades (2 and 3). *p ≤ 0.05, ns, non-significant (*p* = 0.114). Wilcoxon (Mann–Whitney) test; ns, non-significant.

The clinicopathological characteristics of each dog are summarized in Table 1. The correlation between molecular markers in canine UC samples detected by IHC and clinicopathological features was further studied. A statistical analysis revealed a significantly higher expression of MTA1 (*p* = 0.005) and COX2 (*p* ≤ 0.001) in canine UC samples compared to normal control bladder tissues, while, in contrast, E-cad expression was higher in normal tissues compared to UC samples (*p* < 0.001) (Figures 4A–C). An analysis of the relationship between MTA1 and COX2 expression within primary tumor tissues revealed a positive and linear correlation of statistical significance (Figure 4D, *left*). Meanwhile, an analysis of MTA1 and E-cad expression showed a weak inverse association (Figure 4D, *right*).

To evaluate the relationship between the histopathology of UC and the expression of MTA1, we analyzed the differences in MTA1 expression between normal bladder tissues and tumors assigned with different grades using the Meuten and Valli grading systems (Table 1). While all tumor samples were undistinguishably high using the Meuten grade system, we did observe an overall trend for higher MTA1 associated with Valli grade of 3 (Figure 4E, *p* = 0.017). There was no significant correlation between COX2 expression and Valli grades.

**Figure 5:**
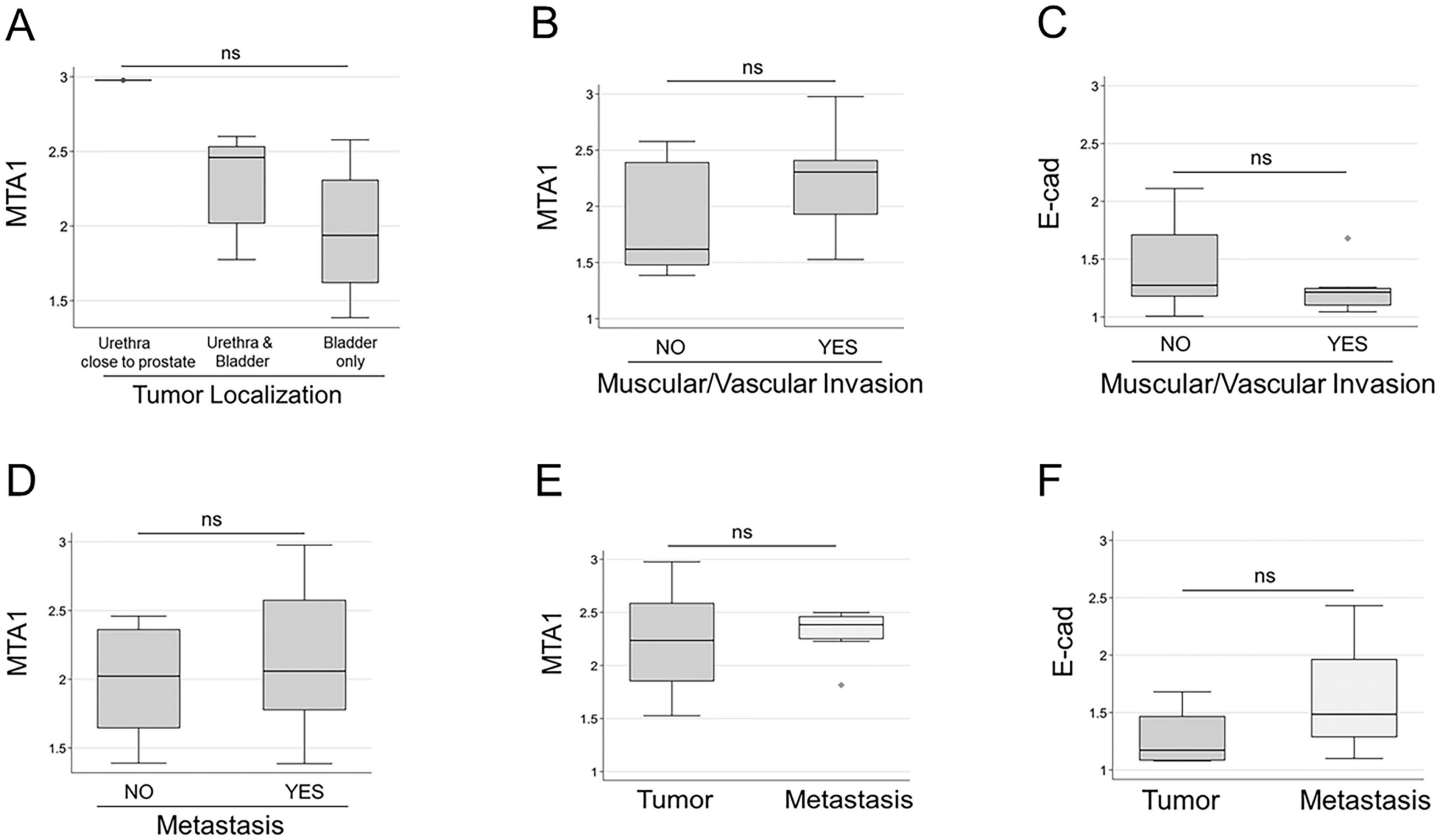
**(A)** MTA1 expression based on tumor localization: prostatic urethra (*n* = 1); urethra close to the bladder (*n* = 5); bladder only (*n* = 17). (ns, *p* = 0.061), Kruskal-Wallis test (non-parametric). **(B)** MTA1 (ns, *p* = 0.359) and **(C)** E-cad (ns, *p* = 0.219) expression in tumors with muscular/vascular invasions and without, Wilcoxon rank-sum (non-parametric). **(D)** MTA1 expression in primary tumors with and without metastasis (ns, *p* = 0.449), Wilcoxon rank-sum test. **(E)** MTA1 expression in eight matched primary tumor-metastasis pairs (ns, *p* = 0.641), Wilcoxon signed-rank test. **(F)** E-cad expression in four matched primary tumor-metastasis pairs (ns, *p* = 0.125), Wilcoxon signed-rank test; ns, non-significant.

Furthermore, we found that MTA1 values varied depending on tumor location. The highest MTA1 expression was detected when the tumor was in the urethra close to the prostate (unfortunately, we had only one sample of prostatic urethra). Medium MTA1 values were detected in tumors located in both the bladder and urethra, while tumors located only in the bladder showed the lowest MTA1 expression (*p* = 0.061) (Figure 5A).

As expected, there was a correlation between Ki67 and mitotic counts among the samples but MTA1 expression did not show statistically significant correlation with either Ki67 or CD31 (data not shown).

All tumors presented with local lamina propria invasion, but some had additional invasion of surrounding muscle and vasculature. To evaluate the role of MTA1- and E-cad -associated tissue invasion, we analyzed their levels in primary tumors with muscle/vascular invasion and those without (Table 1). Results showed a trend for high MTA1 expression in primary tumors with muscle/vascular invasion, whereas a lower MTA1 expression was associated in those tumors with only lamina propria invasion (Figure 5B). In contrast, there was a trend for lower E-cad expression in tumors with muscular/vascular invasion compared to tumors without invasion (Figure 5C).

To evaluate MTA1 expression as a marker for tumor aggressiveness and metastasis in UC, we compared MTA1 expression in primary tumors from dogs without metastasis with samples from dogs with metastasis (Table 1). Albeit statistically non-significant, results followed a trend (*p* = 0.449) towards higher MTA1 values in tumors with metastasis (Figure 5D). Despite positive correlation between MTA1 and COX2 expression in primary tumors, there were no significant differences observed between tumors with or without metastasis when MTA1 and COX2 were considered together (not shown). An analysis of eight matched primary tumor-metastasis pairs showed a trend towards higher MTA1 values in metastatic lesions compared to their primary tumors (Figures 5E, *p* = 0.641). Intriguingly, an analysis of four matched tumor-metastasis pairs all showed higher metastatic E-cad values compared to its primary tumor (*p* = 0.125) (Figure 5F). Overall, these results suggest the tumor- promoting role of MTA1/COX2 in canine UC and its possible but complex association with E-cad-mediated events.

### 3.3 Expression of MTA1 and associated onco-markers in UC cell lines

**Figure 6:**
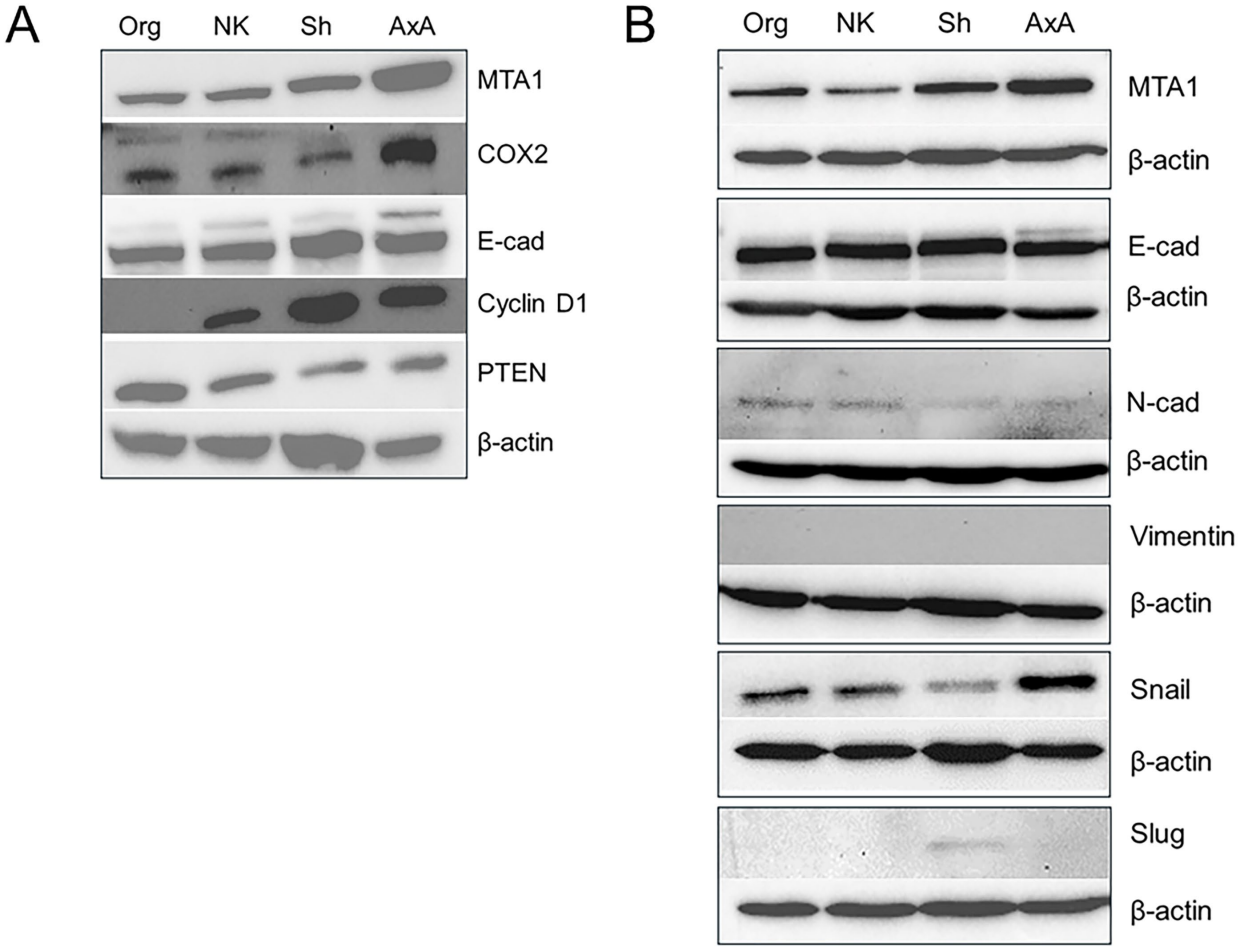
**(A)** Differential expressions of MTA1, COX2, E-cad, Cyclin D1, and PTEN in various canine UC cell lines. **(B)** MTA1 and EMT-associated markers. Western blot analyses were performed at least three times with independent samples. β-actin was used as a loading control.

To be able to address the functional role of MTA1 in canine UC for future studies, we firstly characterized four canine UC cell lines (K9TCC) kindly provided by Dr. Knapp’s group at Purdue University (40, 41). Western blot results are shown in Figure 6. Strong expression of MTA1 was detected in AxA and Sh cells while Org and Nk cells had lower amounts of MTA1. Since MTA1 is associated with cell metastatic characteristics such as invasion and migration (42, 43), high expression of MTA1 was expected in more aggressive cell lines. Indeed, according to Dhawan et al. (41), AxA and Sh cells formed numerous colonies in soft agar assays compared to minimal numbers or no colonies formed by Org and Nk cells, respectively. The expression of COX2 was also highest in AxA cells and lower, yet similar, expression was found across the other cell lines. The expression of a proliferative marker Cyclin D1, which was determined by us previously as a MTA1-associated gene in humans (32, 44), showed a similar trend to that of MTA1 in canine cell lines: it was highest in AxA and Sh aggressive cell lines, while it was low in Nk cells and undetectable in Org cells (Figure 6A). The pattern of PTEN tumor suppressor was also in keeping with its known inverse relationship with MTA1 (32, 45). AxA and Sh express much lower PTEN compared to Nk and Org (Figure 6A).

As our interest was to determine the link between MTA1 and EMT-associated events in canine UC, we analyzed EMT-markers in these cell lines. Interestingly, all cell lines expressed high and comparable levels of E-cadherin, which is typically associated with the epithelial phenotype, and low levels of N-cadherin, a mesenchymal-like marker (46)(Figure 6B). Other EMT-inducers such as transcriptional factors Snail and Slug, known to negatively control E-cad expression (47, 48), were also found in these cells to varying degrees. However, we were unable to detect Vimentin in any of these cell lines with commercially available antibodies (Supplementary Table S1).

These data emphasize the similarities and differences between canine heterogeneous tumor tissues and established cell lines and give us a platform from which to study pertinent signaling pathways that can become targets for therapeutic interventions.

## 4 Discussion

The leading type of canine UC is high-grade muscle-invasive type, comprising of greater than 90% of cases (5). Despite some advances in targeted therapies, the survival rate for invasive UC in dogs remains low: the average survival time for dogs with bladder involvement is about 1 year when chemotherapy is added to COX2 targeted therapy (49). If the prostate or urethra is involved, the average survival time is less. Identifying new molecular targets underlying canine UC progression will help us to develop more efficacious therapies. Here, we showed, for the first time, a high expression of tumor-promoting MTA1 in canine UC clinical samples and UC cell lines. In the current study, we found significantly higher levels of MTA1 in primary tumor tissues and metastatic sites compared to normal bladder tissues. We also demonstrated a strong positive association between high levels of MTA1 and COX2 in UC samples, and a possible involvement of MTA1 in EMT-related events in canine UC. In addition, for the first time, we detected MTA1 expression in canine UC cell lines. Our study indicates that MTA1 may contribute to the progression of canine UC via an association with COX2 and a regulation of EMT- related events.

We found robust differences in the expression of MTA1, COX2 and E-cad between clinical samples of UC and normal bladder. As expected, MTA1 and COX2 expression were higher in UC tumor tissues compared to control bladder tissues, while E-cad expression was significantly lower. Interestingly, MTA1 expression showed dependence on tumor localization. Because of the anatomical uniqueness of the prostate located near the neck of the urinary bladder of male dogs, the location of the UC tumor may have distinctly different primary sites: bladder, urethra or prostatic urethra. This creates difficulties in differentiating between urinary bladder UC and prostate cancer in dogs. Importantly, dogs presenting with prostatic involvement generally have a poorer prognosis and shorter median survival time (50). Unfortunately, we had only one sample of prostatic urethra, but it showed the highest MTA1 expression. Studies with more prostatic urethra samples will follow to confirm the role of MTA1 in more aggressive canine UC.

Although we did not detect a statistical difference in MTA1 overexpression between primary tumors that developed metastasis and those that did not, matched tumor-metastasis pairs showed a tendency for higher MTA1 in lung metastatic lesions, suggesting that MTA1 might work as a prognostic marker. In fact, MTA1 has been associated with poor prognosis of many types of cancers (51–55). Due to an absence of canine gene expression databases, we searched a publicly available human database for an analysis of the prognostic significance of MTA1 in bladder cancer. We found that in a microarray study of 60 tissue samples (56), MTA1 mRNA expression was significantly higher in primary tumor tissue (*n* = 33) compared to normal urothelium (*n* = 14), and that the expression was even higher in cases involving muscle invasion (*n* = 13) (Supplementary Figure S2). Considering the limited number of cases in our study, a large-scale investigation must be conducted to clarify the prognostic value of MTA1 in canine UC.

While MTA1 is a “new” molecule in canine UC, the inducible COX2 inflammatory and oncogenic pathway that participates in cancer cell proliferation, migration, survival, stimulation of angiogenesis and promotion of drug resistance has already been recognized in canine and human invasive UC (57, 58). In fact, COX2 inhibitors (i.e., piroxicam and celecoxib) administered alone and in combination with chemotherapy (7–9, 59, 60), are commonly used clinically. We found that there is a positive and statistically significant linear correlation between MTA1 and COX2 in our clinical samples of canine UC, with a similar correspondence among UC cell lines *in vitro*. While literature establishing a link between MTA1 and COX2 is sparse, one publication demonstrated a direct link between these biomarkers in human lung cancer (61). With this in mind, MTA1 inhibitors combined with COX2 inhibitors might substantially improve antitumor efficacy.

The role of MTA1 in cancer cell EMT-related events has been shown previously (27, 28). Several studies have demonstrated that miRNA-mediated targeting of MTA1 resulted in repression of EMT leading to diminished invasion and migration of human pancreatic, gastric, and non- small cell lung cancer (62–64). We, too, have shown an inverse correlation between MTA1 and E-cad in human prostate cancer (31, 32).

In the clinical samples used in our current study, E-cad expression was significantly lower in canine UC tissues compared to normal urothelium, which was also previously reported in a study involving two canine UC cases with plasmacytoid and rhabdoid features (65). Furthermore, when we compared E-cad levels in primary tumors that invaded muscle with tumors that did not, we detected a downregulation of E-cad in tumors with invasion (Figure 5C). However, we were surprised to discover that the mean E-cad strong staining intensity (+++) IHC score was significantly higher in metastatic lesions compared to primary UC tissues. This finding was recapitulated when four matched pairs of primary tumor and lung metastasis showed elevated levels of E-cad in metastatic tumor cells compared to primary tumor cells (*p* = 0.125) (Figure 5F). In addition, we detected high levels of E-cad in all canine UC cell lines tested. Though unexpected, our results are in accordance with a recently published comprehensive review article on the status of E-cad in carcinoma tissues and cell lines (66), in which authors assert that “the role of E-cad in tumor progression and metastasis may have been oversimplified” over the years. The authors present a thorough analysis from multiple large datasets on clinical cancer samples showing that levels of E-cad mRNA and protein are elevated during tumor progression and remain elevated in most metastatic sites. Moreover, they also found that most of the carcinoma cell lines (epithelial cells) express elevated levels of E-cad (66).

The association between MTA1 and E-cad expression was analyzed in our clinical samples. Results showed a trend towards an inverse relationship between MTA1 and E-cad. These data are in good agreement with an independent study of α4 regulated E-cad in bladder urothelial carcinoma in humans, in which a significant inverse correlation was shown between E-cad and MTA1 in 187 clinical samples (67).

In summary, to our knowledge, this study is the first to report on MTA1 overexpression in canine UC and its potential as a therapeutic target for high-grade invasive UC in dogs.

A key limitation of our study is the lack of sample size, resulting in some statistically non-significant trends. Our results provide a scientific rationale for further clinical and mechanistic studies on the functional role of MTA1 in canine UC. A greater number of clinical samples including metastatic lesions would be needed to establish the clinical significance of high MTA1 as a prognostic factor and potential target in canine UC. Functional studies are already underway to establish the role of MTA1-mediated EMT events in canine UC progression. Since MTA1 is closely associated with tumor aggressiveness and metastasis in cancer, it may be considered as a possible interceptive and therapeutic target for canine invasive UC treatment.

We identified MTA1 as a novel potential molecular marker and target in canine UC. Our data suggest that MTA1 may play an essential role in the progression of UC in dogs, particularly through an association with COX2 and by facilitating EMT events. However, further studies are warranted for elucidating the exact molecular mechanisms responsible for MTA1-mediated progression of canine UC.

## Data Availability

The original contributions presented in the study are included in the article/Supplementary material, further inquiries can be directed to the corresponding author.

## Glossary

AKT: V-akt murine thymoma viral oncogene (protein kinase B)
ANOVA: Analysis of variance
ATCC: American Type Culture Collection
AxA: K9TCC-PU-AxA cell line
CD31: Cluster of differentiation 31
CIS: Carcinoma *in situ*
COX2: Cyclooxygenase 2
DMEM/F12: Dulbecco’s Modified Eagle Medium/Nutrient Mixture F-12
E-cad: E-cadherin
EGFR: Epidermal growth factor receptor
EMT: Epithelial-to-mesenchymal transition
H&E: Hematoxylin and eosin
HER2: Human epidermal growth factor 2
IHC: Immunohistochemistry
K9TCC: Canine transitional cell carcinoma cell line
Ki67: Cellular protein marker of proliferation
LIU: Long Island University
MTA1: Metastasis-associated protein 1
mTOR: Mammalian target of rapamycin
Nk: K9TCC-PU-Nk cell line
NS: Non-significant
Org: K9TCC cell line
PD-1: Programmed cell death protein 1
PI3K: Phosphoinositide 3-kinase
PTEN: Phosphatase and tensin homolog
RTK: Receptor tyrosine kinase
SEM: Standard error of mean
Sh: K9TCC-PU-Sh cell line
TCC: Transitional cell carcinoma
UC: Urothelial carcinoma
