# Supplementary material for: Identification of metastasis-associated protein 1 (MTA1) as a new molecular marker for canine urothelial carcinoma
Source: Front Vet Sci. 2025 Apr 25;12:1527167. doi: 10.3389/fvets.2025.1527167 (PMC12062746; doi:10.3389/fvets.2025.1527167)
Supplement: Supplementary file 1 [file Data_Sheet_1.pdf]

Supplementary Data, Campanelli et al
Manuscript title: Identification of Metastasis-Associated Protein 1 (MTA1) as a New Molecular Marker for Canine Urothelial Carcinoma
Manuscript ID: 1527167

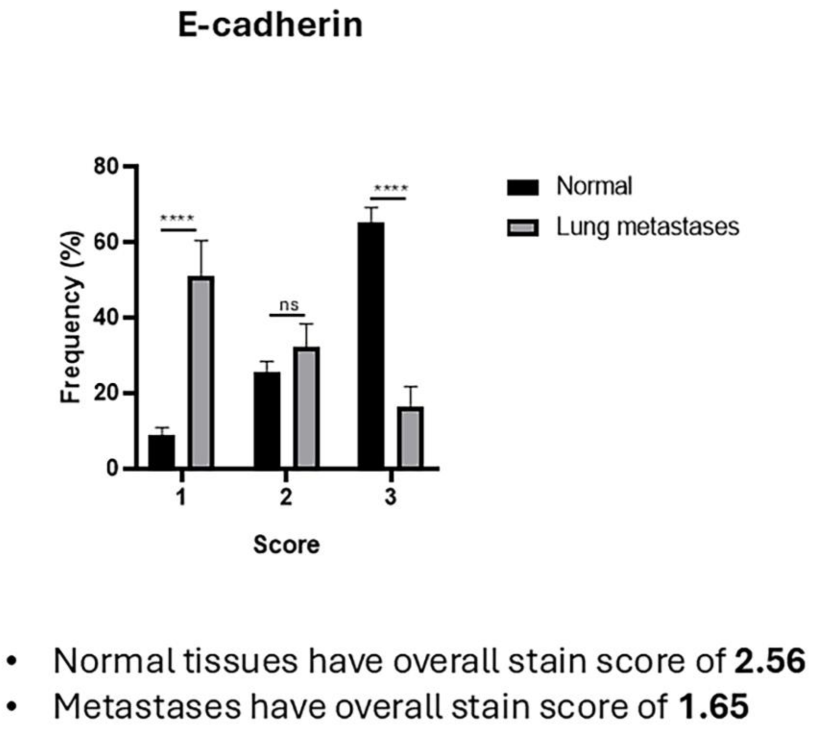

Figure S1. The stain scores for E-cad expression in metastasis compared to normal tissues. Values are mean ± SEM. ****$p < 0.0001$; ns, non-significant (multiple *t*-tests).

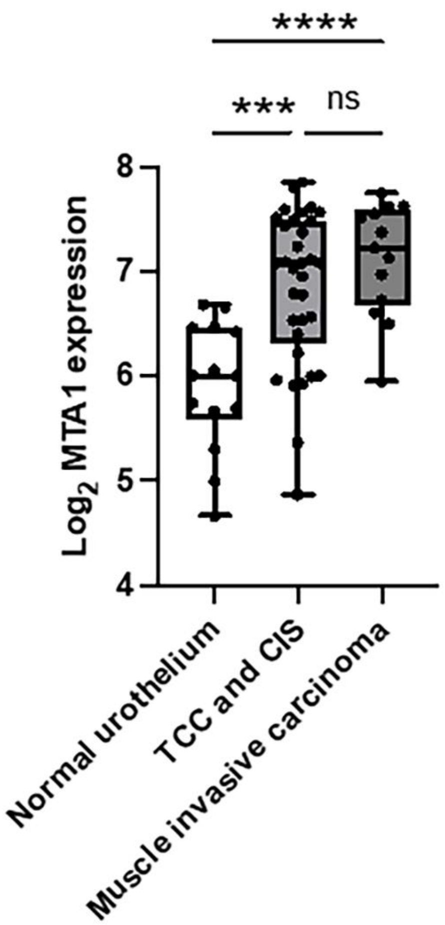

Figure S2. Graph depicting MTA1 gene expression in human bladder samples (n = 60) analyzed from microarray data reported to Gene Expression Omnibus (GEO) database (Accession no: GSE3167, ID: 14786580) [49]. A statistically significant increase in MTA1 gene expression was observed in local transitional cell carcinoma and carcinoma in situ (n = 33) compared to normal urothelium (n = 14), with a trend towards a further increase in expression in aggressive muscle invasive carcinoma (n = 13). Values are median and min/max data points. ***p < 0.001; ****p < 0.0001; ns, non-significant (One-way ANOVA).

Table S1. Antibodies used for IHC and western blot

Antibody	Application	Dilution	Source	Catalog#
β-actin	WB	1:2500	Santa Cruz Biotechnology	sc-69879
CD31	IHC	1:40	Dako - Agilent Technologies	M0823
COX2	WB<br>IHC	1:2000<br>1:100	Cayman Chemical Company	160107
Cyclin D1	WB	1:1000	Cell Signaling Technologies	2922
E-cadherin	WB<br>IHC	1:1000<br>1:400	Cell Signaling Technologies	3195
Ki67	IHC	Ready-to-Use	Dako - Agilent Technologies	GA626
MTA1	WB<br>IHC	1:1000<br>1:50	Cell Signaling Technologies	5647
N-cadherin	WB	1:500	Cell Signaling Technologies	13116
PTEN	WB	1:1000	Cell Signaling Technologies	9188
Slug	WB	1:500	Cell Signaling Technologies	9585
Snail	WB	1:500	Cell Signaling Technologies	3879
Vimentin	WB<br>WB	1:2000<br>1:1000	GeneTex<br>Cell Signaling Technologies	GTX100619<br>5741
